# HPV‐vaccination and cancer cervical screening in 53 WHO European Countries: An update on prevention programs according to income level

**DOI:** 10.1002/cam4.2048

**Published:** 2019-04-16

**Authors:** Emma Altobelli, Leonardo Rapacchietta, Valerio F. Profeta, Roberto Fagnano

**Affiliations:** ^1^ Department of Life, Health and Environmental Sciences University of L'Aquila L'Aquila Italy; ^2^ Epidemiology and Biostatistics Unit Local Health Unit‐ Teramo University of L'Aquila L'Aquila Italy; ^3^ Epidemiology and Biostatistics Unit Local Health Unit Teramo Italy; ^4^ Local Health Unit Teramo Italy

**Keywords:** cervical cancer, coverage, HPV vaccination, income level, screening programs, surveillance

## Abstract

Human papillomavirus (HPV) is the most common sexually transmitted disease in the world. The aim of our study is to describe the differences in HPV‐vaccination coverage and screening programs in WHO European Countries notably according to income levels. Multiple correspondence analysis was applied to examine the association among the following variables: Gross National Income (GNI) levels (Lower‐Middle Income, LMI; Upper‐Middle Income, UMI; and High Income, HI); type of CC screening program (coverage; opportunistic/organized); vaccination payment policies (free or partial or total charge); mortality rates/100 000 (≤3; >3‐6; >6‐9; >9); incidence rates/100 000 (≤7; >7‐15; >15‐21; >21). Data HPV‐vaccination start (years) (2006‐2008; 2009‐2011; 2012‐2014; >2014; no program); coverage HPV‐vaccination percentage (≤25; 26‐50; 51‐75; >75); data screening start (years) (<1960; 1960‐1980; 1981‐2000; >2000); primary screening test (HPV, cytology), and screening coverage percentage (≤25; >25‐50; >50‐75; >75). A high income is associated with: start of screening before 1960, medium‐high screening coverage, organized screening, start of vaccination in the periods 2009‐2011 and 2012‐2014 and high immunization coverage. On the other hand, lower‐middle income is associated with: late start of vaccination and screening programs with cytology as primary test, high mortality and incidence rates and lower‐medium vaccination coverage. Our results show a useful scenario for crucial support to public health decision‐makers. Public health authorities should monitor the HPV‐vaccinated population in order to determine more precisely the effects on short‐ and long‐term incidence and mortality rates. In fact, the greater the vaccination coverage, the greater will be the efficacy of the program for the prevention of CC and other HPV‐related diseases.

## 1 INTRODUCTION

Human papillomavirus (HPV) is the most common sexually transmitted disease in the world.^1^ The persistent infection with high‐risk HPV causes Cervical Cancer (CC).^2^ In female population it is the fourth cancer and the second most common from 25 to 40 years of age.^3^ Strategies against HPV infection are vaccination and safe sex education.^4^ Countries that have performed HPV‐vaccination programs have showed a decrease in the prevalence in the population of the HPV 16, 18 genotypes.^5^ HPV‐related disease incidence and mortality are the most common measures used to evaluate the impact of vaccination in European Countries.^6^ In Europe, HPV‐vaccination coverage rates vary from 30% to 80% with school‐based programs.^7^ Information campaigns of health interventions are closely linked to the success of a vaccination program. In fact, the greater the vaccination coverage, the greater will be the efficacy of the program for the prevention of CC and other HPV‐related diseases.^8^ In 2006, the European Medicines Agency (EMA) endorsed the quadrivalent HPV vaccine, in 2007 the bivalent, while in June 2015 a 9‐valent vaccine was recommended.^9^

It is important to underline that the two primary (HPV vaccination) and secondary strategies (screening, early diagnosis) will lead to the reduction of incidence and mortality for CC.^10^ Relatively to Europe, with regard to CC, vaccination and screening programs show differences among Countries; indeed, relatively to screening, there are organized and nonorganized (opportunistic) programs.^11^ Knowledge of the onset of CC, new technologies, HPV test as primary screening test^11^ along with home self‐sampling^12^, ^13^, ^14^ modified screening programs in many European Countries.^15^

Cervical cancer screening programs together with primary prevention could contribute to reducing social inequalities between central and eastern European Countries.^16^

The aim of the study was to describe the differences in HPV‐vaccination coverage and screening programs in WHO European Countries notably according to income levels.

## 2 MATERIALS AND METHODS

### 2.1 Gross national income (GNI)

**Figure 1:**
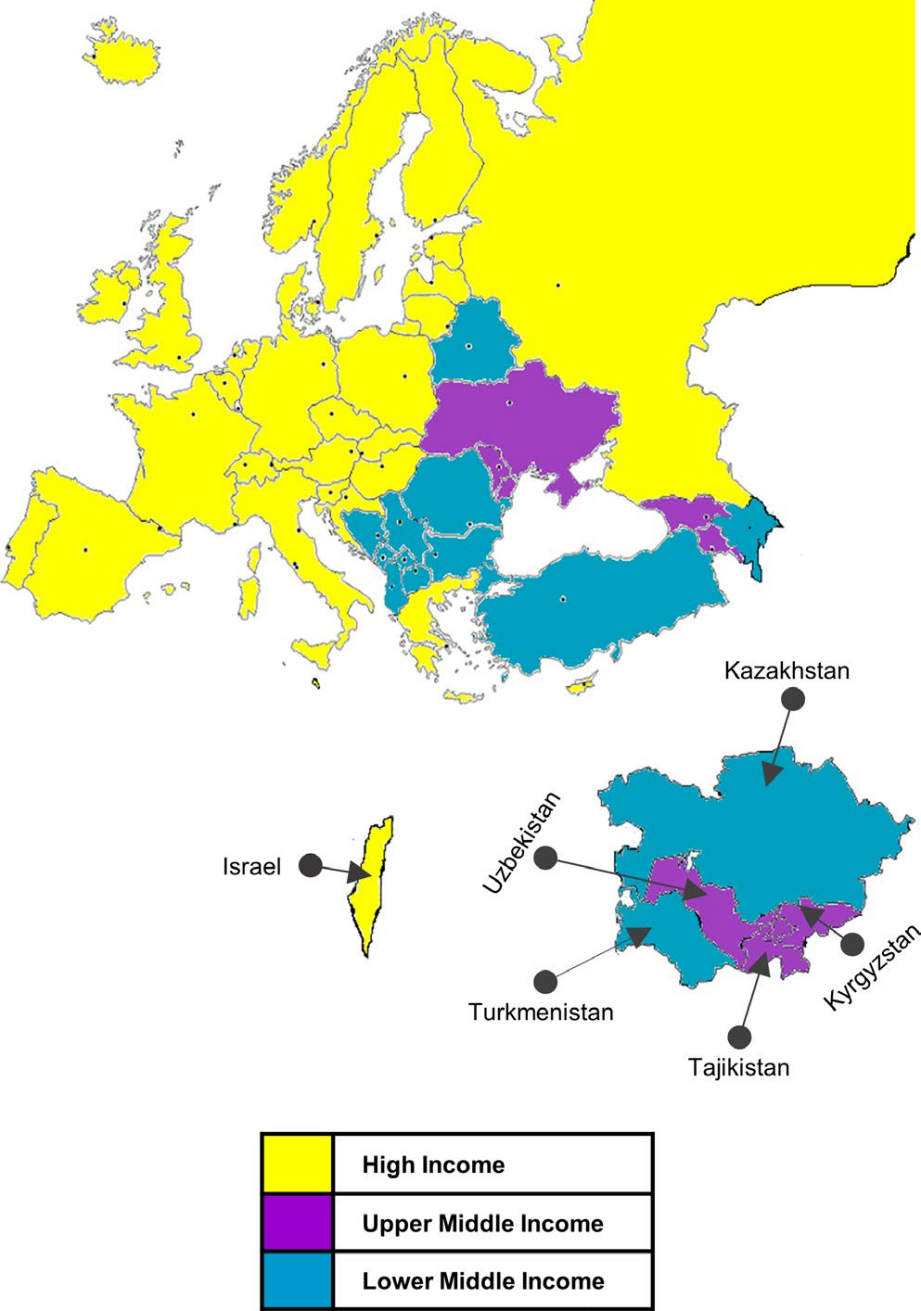
Map of Countries grouped according to income levels

According to the World Bank, economies can be divided into low income (LI), lower‐middle income (LMI), upper‐middle income (UMI), and high income (HI) in relation to GNI per capita^17^ (Figure 1). In this study, the 53 WHO ER Countries were thus divided into: LMI, $1026‐4035 (Armenia, Georgia, Kyrgyzstan, Moldova, Tajikistan, Ukraine, and Uzbekistan); UMI, $4036‐12 475 (Albania, Azerbaijan, Belarus, Bosnia and Herzegovina, Bulgaria, Kazakhstan, FYR of Macedonia [FM], Hungary, Montenegro, Romania, Serbia, Turkey, and Turkmenistan); and HI, $12 476 (Austria, Belgium, Czech Republic, Denmark, Estonia, Finland, France, Germany, Greece, Iceland, Ireland, Israel, Italy, Luxembourg, Norway, Poland, Portugal, Slovakia Republic, Slovenia, Spain, Sweden, Switzerland, the Netherlands, and the United Kingdom, Andorra, Croatia, Cyprus, Malta, Monaco, Latvia, Lithuania, Russian Federation, and San Marino) (World Bank and Lending Groups 2016) (Table 1).^18^, ^19^, ^20^, ^21^, ^22^, ^23^, ^24^, ^25^, ^26^, ^27^, ^28^, ^29^, ^30^, ^31^, ^32^, ^33^, ^34^, ^35^, ^36^, ^37^

**Table 1:** Differences of CC burden, primary and secondary prevention programs in 53 Countries of the WHO Region

Country	National immunization			Cancer screening						
	Incidence 100.000	Mortality 100.000	Date start Age at beginning	Policy payment Coverage% (year)	Organization Start date	Regions Coverage % (year)	Primary Test	Age target Age	Screening Interval Years	Payment Policy
	2008 2012	2008 2012								
Austria	5.7 5.8	2.2 2.6	2014 9‐12	Fully covered by patient NR	Opportunistic 1970	National 86.6 (2014)	Cytology PAP	>18	1	Free of Charge
Andorra	NR NR	NR NR	2014 12	NR NR	Opportunistic NR	NR 61.4 (2011)	Cytology PAP	>18	1	NR
Belgium	8.4 8.6	2.5 2.3	2007 12	75% supported by national health authorities NR	Opportunistic 1965 Organized 1994	Regional 68.7 (2013)	Cytology PAP	25‐64	3	Free of Charge
Croatia	11.8 10.0	4.2 3.4	2016 NR	Fully covered by national health authorities^23^ NR	Opportunistic 1960 Organized 2012	National 65.3 (2003)	Cytology PAP^a^	25‐64	3	Free of Charge
Cyprus	4.5 4.1	2.6 1.3	2016 11‐12	NR NR	Opportunistic NR	NR 67.4 (2012)	Cytology PAP	24‐65	NR	NR
Czech Republic	14.0 14.1	4.6 4.8	2012 13	Covered by general health insurance for routine 81.0 (2011)	Opportunistic 1947 Organized 2008	National 87.2 *(*2014*)*	Cytology PAP	25‐60	1	Free of Charge
Denmark	12.1 10.6	3.1 2.6	2009 12	Fully covered by national health authorities 79.0 (2011)	Opportunistic 1962 Organized 2006	National policy, local implementation 64.1 *(*2014*)*	HPV Cytology PAP^12^	60‐64 HPV^b^ 23‐59 Cytol.	5 (60‐64) HPV 3 (23‐59) Cytology	Free of Charge
Estonia	15.8 19.9	7.3 7.5	No program	‐	Organized 2006	National 57.7 *(*2014*)*	Cytology PAP^12^	30‐59	5	Free of Charge
Finland	4.5 4.3	1.5 1.3	2013 11‐12	Fully covered by national health authorities 29.3 (2012)	Organized 1963	National 69.0 *(*2015*)*	HPV Cytology PAP^12^	30‐64	5^c^	Free of Charge
France	7.1 6.8	1.9 1.8	2007 11‐14	65% supported by national health authorities 24.0 (2008)	Organized 1991	Regional^12^ 75.4 (2014)	Cytology PAP	25‐65	3	Insurance Copayment
Germany	6.9 8.2	2.6 2.6	2007 9‐14	Fully covered by national health authorities NR	Organized 1971 (west) 1991 (expanded to the eastern country)^17^	National 80.4 (2014)	Cytology PAP	≥20	1	Free of Charge
Greece	4.1 5.2	1.4 1.9	2008 11‐15	Fully covered by national health authorities NR	Opportunistic 1991	National 75.5 (2014)	Cytology PAP	≥20	1	NR
Hungary	16.6 18.0	6.6 6.2	2014 NR	NR 88.0 (2014)	Opportunistic 1950 Organized 2003	National 40.1 (2015)	Cytology PAP	25‐65	3	Free of Charge
Iceland	8.4 7.9	0.8 1.9	2011 12	Fully covered by national health authorities 84.0 (2013)	Organized 1964^20^	National 71.0 (2015)	Cytology PAP^12^	20*‐*69	2 (20‐39) 4 (40‐69)	NR
Ireland	10.9 13.6	3.6 4.0	2010 12‐13	Fully covered by national health authorities 84.9 (2014)	Organized 2008	National 78.7 (2015)	Cytology PAP	25‐60	3 (25‐44) 5 (45‐60)	Free of Charge
Israel Outside European institutions	NR 4.6	2.4 2.0	2013 13	NR 80.0 (2011)	Opportunistic NR	Regional 32.0 (2008)	Cytology PAP	25‐65^21^	3	Free of Charge^31^
Italy	6.7 6.7	0.8 0.9	2007 12	Fully covered by national health authorities 65.0 (2011)	Organized 1996	National policy, local implementation^14^ 79.0 *(*2015*)*	HPV Cytology PAP	25‐64	5 HPV 3 Cytology	Free of Charge
Latvia	12.4 17.3	6.9 8.5	2010 12	Fully covered by national health authorities NR	Opportunistic 1960 Organized 2009	National 25.2 (2016)	Cytology PAP^18,^ d	25‐70	3	Free of Charge
Lithuania	21.0 26.1	10.6 9.0	2016 NR	NR 29.0 (2009)	Organized 2004	National 74.0 (2014)	Cytology PAP	25‐60	3	Free of Charge
Luxembourg	6.3 4.9	5.6 1.7	2008 12‐18	Fully covered by national health authorities 17.0 (2009)	Opportunistic 1962 Organized 1990	National 83.6 (2014)	Cytology PAP	>15	1	NR
Malta	2.1 3.8	2.1 2.4	2012 12	Fully covered by national health authorities NR	Opportunistic NR	National 49.3 (2008)	HPV Cytology PAP	>30 HPV 25‐50 Cytol.	5 HPV 3 Cytology^e^	Free of Charge
Monaco	NR NR	NR NR	2011 14	NR NR	Opportunistic NR	NR NR	Cytology PAP	21‐65	1^f^	NR
Norway	9.4 9.8	3.0 2.1	2009 12	Fully covered by national health authorities 63.0 (2011)	Opportunistic 1970^12^ Organized 1995^12^	National 74.1 (2015)	Cytology PAP^12,^ g	25‐69	3	NR
Poland	11.6 12.2	7.3 6.7	No Program	‐ *‐*	Opportunistic 1970 Organized 2006	National 21.2 (2013)	Cytology PAP	25‐59	3	Free of Charge
Portugal	12.2 9.0	3.4 2.8	2008 13	Fully covered by national health authorities 84.0 (2011)	Organized Central Region 1990 Alentejo Region 2008	Regional 70.7 (2014)	Cytology PAP	25‐64	3	Free of Charge
Russian Federation Outside European institutions	13.3 15.3	6.6 6.9	Partial program 2009 12‐13	NR NR	Organized NR	NR 72.0 (2012)	Cytology PAP	>18	1	NR
San Marino	NR NR	NR NR	2008 11‐14	Fully covered by national health authorities NR	Opportunistic 1968 Organized 2006^33^	National 82.0 *(*2017*)* ^19^	HPV Cytology PAP	30‐65 HPV 25‐30 Cytol.	5 HPV 3 Cytology	NR
Slovakia Republic	15.8 16.1	6.5 6.9	2014 12	NR 55.0 (2012)	Opportunistic 1980 Organized 2008	National 69.0 (2014)	Cytology PAP	23‐64	1	Free of Charge
Slovenia	11.1 10.5	3.1 2.9	2009 11	Fully covered by national health authorities 70.8 (2012)	Opportunistic 1960 Organized 2003	National 71.9 (2016)	Cytology PAP	20‐64	3	Free of Charge
Spain	6.3 7.8	2.1 2.1	2007 11‐14	Fully covered by national health authorities 78.5 (2010)	Organized 1993	National^18^ 72.7 (2014)	HPV Cytology PAP	30‐65 HPV 25‐65 Cytol.	5 HPV 3 Cytology	Free of Charge
Sweden 4,100,000	7.8 7.4	2.2 2.8	2010 10‐12	Fully covered by national health authorities 82.0 (2012)^24^	Opportunistic 1950 Organized 1967	National 81.7 (2015)	HPV Cytol. PAP^12,^ h	30‐64 HPV 23‐29 Cytol.^12^	3 (30‐50) 7 (51‐64) HPV. 3 (23‐29) Cytology	Free of Charge
Switzerland	4.0 3.6	1.4 1.5	2008 11‐14	NR NR	Opportunistic NR	NR 74.5 (2012)	Cytology PAP	>20	3	Insurance Copayment
Netherlands	6.8 6.8	2.3 1.9	2010 12	Fully covered by national health authorities 79.5 (2014)	Opportunistic 1970 Organized 1980	National 64.4 (2015)	HPV^i^ Cytology PAP	30‐60	5 HPV 5 Cytology	Free of Charge
United Kingdom	7.2 7.1	2.4 2.2	2008 12‐13	Fully covered by national health authorities 91.4 (2013)	Opportunistic 1964 Organized 1988	National 77.5 (2016)	Cytology PAP^j^	25‐64	3 (25‐49); 5 (50‐64)	Free of Charge
Albania	7.1 5.0	1.5 1.8^3^	No Program	NR NR	Opportunistic NR	NR 2.7 (2002)	Cytology PAP	>20	2‐3	NR
Azerbaijan Outside European institutions	NR 9.8	NR 3.9^3^	No Program	NR NR	No Program	1.1 (2001)	Acetic acid visualization VIA	NR	‐	NR
Belarus Outside European institutions	13.2 13.2	6.2 4.7^3^	No Program	NR NR	Opportunistic NR	NR 75 (2015)	Cytology PAP	>18	1	NR
Bosnia and Herzegovina	9.1 13.7	NR 2.7	No Program	NR NR	Organized NR	National 39.8 (2003)	Cytology PAP	21‐70	1	NR
Bulgaria	21.9 24.5	7.0 7.8	2012 12	Covered by general health; catch‐up is opportunistic and not free of charge NR	Opportunistic NR	NR 46.8 (2008)	Cytology PAP	30‐59	3	NR
Kazakhstan Outside European institutions	NR 29.4	8.8 9.1	Partial program 2013 11	NR NR	Organized NR	National 80.3 (2003)	Cytology PAP	30‐60	5	NR
FRY of Macedonia	22.0 12.4	4.1 3.5	2009 12	NR NR	Organized 2015	National 60.0 (2015)	Cytology PAP	30‐55	3	NR
Montenegro	13.0 20.2	5.2 5.8^3^	No Program	NR NR	Opportunistic NR	NR NR	Cytology PAP	25‐64	3	NR
Romania	23.9 28.6	13.7 12.0	2008 12‐14	Fully covered by national health authorities NR	Opportunistic 1965 Organized 2012	National 8.1 (2014)	Cytology PAP	25‐64	5	Free of Charge
Serbia	20.9 23.8	10.3 9.3	2017^21^ 12	NR NR	Opportunistic 1960 Organized 2011^22^	National 57.1 (2013)	Cytology PAP	25‐65	3	Free of Charge
Turkey	NR 4.3	NR 1.7	No Program	NR NR	Opportunistic 1985 Organized 2004^23^	National 46.5 (2015)	HPV^23,^ k	30‐65	5	NR
Turkmenistan Outside European institutions	NR 13.1	5.9 10.1	2016 9	NR NR	Opportunistic NR	National NR	Cytology PAP	>20	1	NR
Armenia	NR 13.8	3.7 5.5	No Program	NR NR	Opportunistic NR	NR 9.3 (2010)	Cytology PAP	30‐60	3	NR
Georgia EU19	NR 14.2	NR 5.7	NR	NR 36.2 (2012)	Opportunistic NR	NR 9.0 (2011)	Cytology PAP^l^	25‐60	3	Free of Charge
Kyrgyzstan Outside European institutions	NR 23.7	12.6 11.4	No Program	NR 53.4	Opportunistic NR	NR 10‐50 (2015)	Cytology PAP	NR	5	NR
Republic of Moldova	17.1 19.6	8.6 7.5	NR	NR NR	Organized NR	National 70.0 (2015)	Cytology PAP	>20	2	NR
Tajikistan Outside European institutions	NR 9.9	NR 4.9	NR	NR 65.0 (2012)	Opportunistic NR	NR 10‐50 (2015)	Cytology PAP	>20	NR	NR
Ukraine	NR 16.6	7.4 7.5	No Program	NR 86.7 (2014)	Opportunistic NR	NR 73.7 (2003)	Cytology PAP	18‐65	1	NR
Uzbekistan Outside European institutions	NR 13.5	NR 6.4	Announced 12	NR NR	Opportunistic NR	NR NR	Cytology PAP	25‐49	NR	NR

R: Not Reported.

a Acetic acid visualization VIA HPV secondary test as a triage to borderline cytology and as a follow‐up after treatment of severe cervical lesions.

b Interval between negative screens is three years for women aged 23‐49 and five years for women aged 50‐64. The primary screening test is cytology for women aged 23‐59 with HPV as a triage test. HPV DNA test is primary screening for women aged 60‐64 years.

c Primary screening test is predominantly cytology but can also be HPV. The sample is examined for cell changes (the traditional Pap test) or the Human Papillomavirus. If there is cancer‐related HPV, the screening sample is checked for possible cervical cell changes (Pap test).

d HPV testing is not reimbursed.

e Screening ages: Above 25 (cytology), Above 30 (HPV test). Screening interval: Cytology every 3 years (ages 25‐50), VIA every 5 years (above 50). HPV test every 5 years.

f 1, 3 after 2 consecutive annual negative Cytology test.

g HPV as primary screening test is underway in part of the country for women between 34 and 69 years of age.

h Reflex testing with HPV is done for cytology positive test (ASCUS/LSIL or worse) below the age of 30 and reflex testing with cytology for HR HPV positive test above the age of 30. A double test (cytology and HPV) is recommended for women at age 41. Women with HPV positive/cytology negative tests should repeat screening after 3 years. Women with ASCUS/LSIL (regardless of HPV status) below the age of 28 are not referred to colposcopy, but repeat cytology.

i Replace Pap‐test with hrHPV DNA test as primary screening test (since 2016).

j If slightly abnormal cells are present, the human papillomavirus (HPV) will be tested.

k HPV test since 2015.

l HPV test undergoing project.

### 2.2 Sources of WHO European epidemiological data

The main data source, the GLOBOCAN 2012 website of the International Agency for Research on Cancer (IARC), provides access to several databases that allow assessing the impact of CC in 184 Countries or territories.^38^

These data were supplemented using the literature, ministerial web site of WHO ER Countries, World Bank Open Data Web site and the World Cancer Registry (X edition).^17^

### 2.3 Statistical analysis

Multiple correspondence analysis (MCA) was applied to examine the association among the following variables: GNI levels (LMI, UMI, and HI); type of CC screening program in each country (coverage; opportunistic/organized); vaccination payment policies (free or partial or total charge); mortality rates/100 000 (≤3; >3‐6; >6‐9; >9); incidence rates/100 000 (≤7; >7‐15; >15‐21; >21). Data HPV‐vaccination start (years) (2006‐2008; 2009‐2011; 2012‐2014; >2014; no program); HPV‐vaccination coverage percentage (≤25; 26‐50; 51‐75; >75); data screening start (years) (<1960; 1960‐1980; 1981‐2000; >2000); primary screening test (HPV, cytology); screening coverage percentage (≤25; >25‐50; >50‐75; >75).

These variables were coded as ordinal, nominal or dummy, as appropriate, and incorporated into the model.

## 3 RESULTS

### 3.1 Multiple correspondence analysis

**Figure 2:**
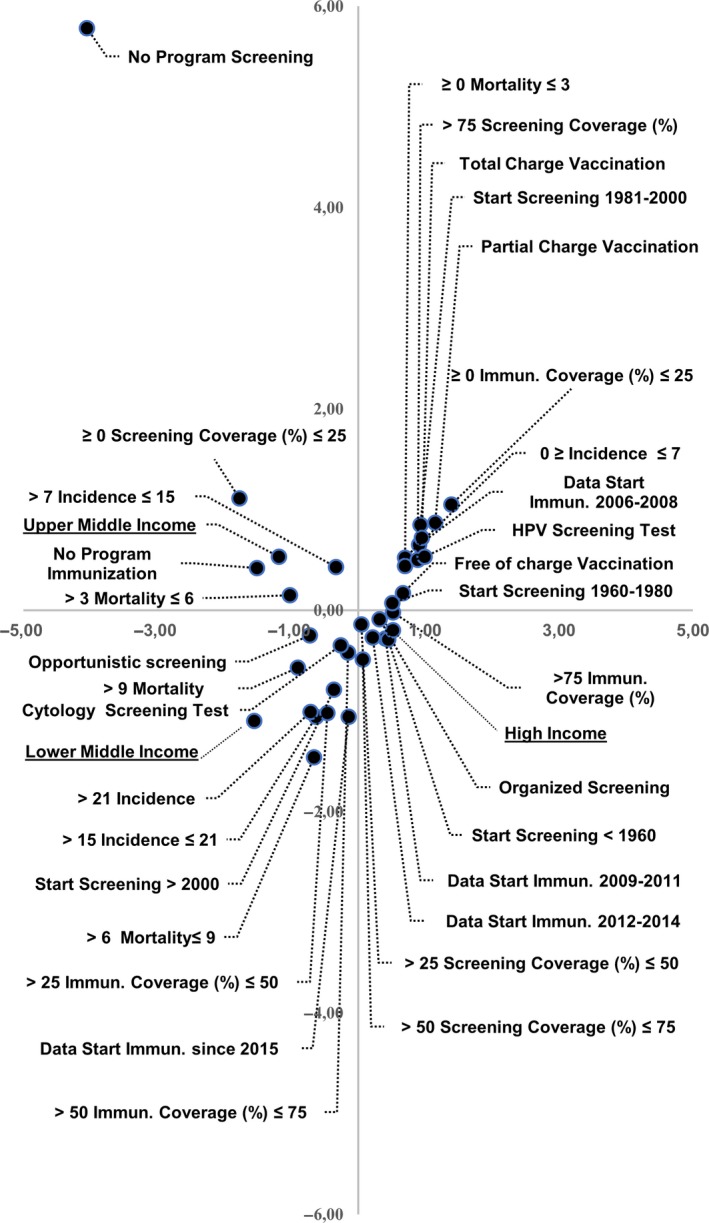
Association among variables included in model of multiple correspondence analysis

**Figure 3:**
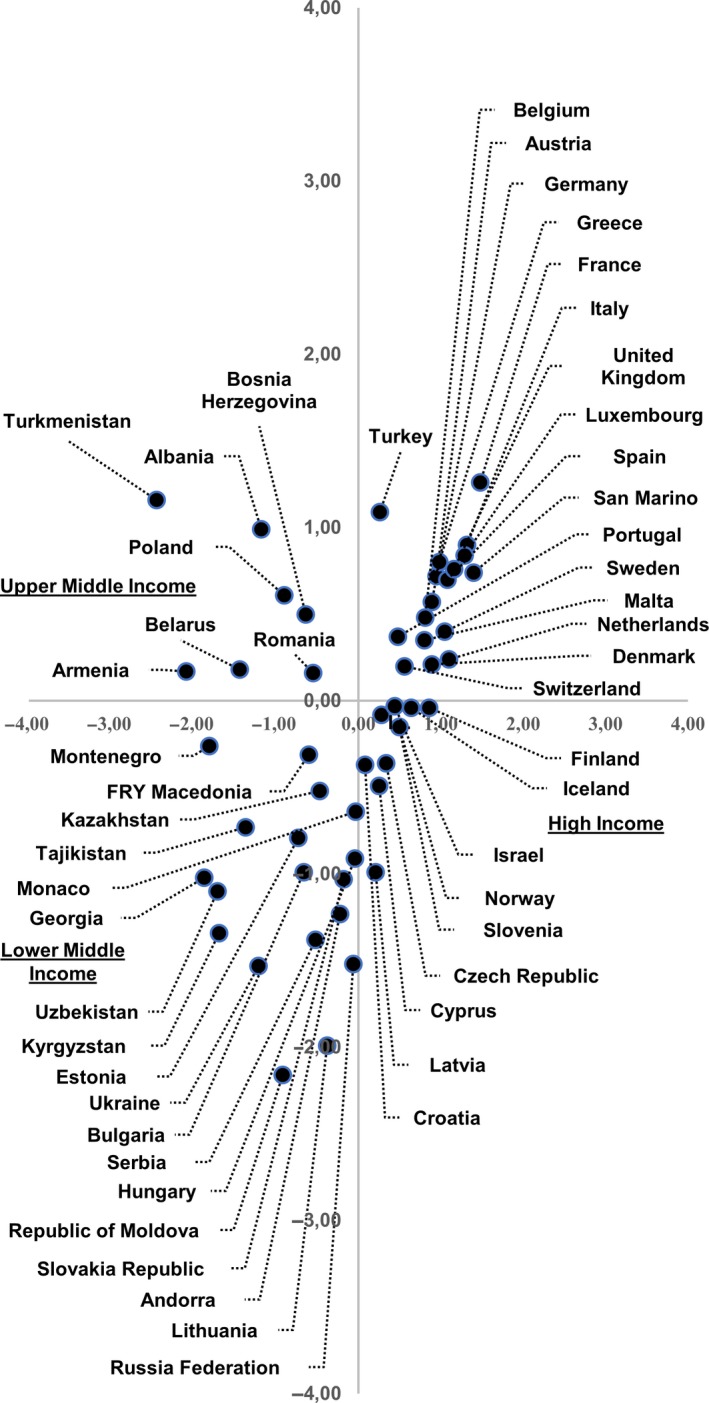
Distribution of 53 European Countries according to multiple correspondence analysis

The results of MCA are shown in Figures 2 and 3. We identified two dimensions that explain 82% of the variance: the first is 49% and the second being 33%.

The first quadrant (top right) identified the following variables: an early initiation of vaccination programs based on HPV screening as primary test; a high‐screening coverage and low incidence and mortality rates. In addition, low‐vaccination coverage and different payment policies (free, partial or total charge) for vaccination programs are located in this quadrant. High income, screening before 1960, medium‐high screening coverage, start of vaccination in the periods 2009‐2011 and 2012‐2014, and high‐immunization coverage are in the fourth quadrant (bottom‐right). On the left side, we can see medium‐low and medium‐high income, low attention to primary and secondary prevention with high rates of occurrence. In the second quadrant (top left), instead, we can observe upper‐middle income, total absence of screening and vaccination programs, medium‐low incidence and mortality rates. The third quadrant (bottom left) stands out with lower‐middle income, late start of vaccination programs and screening with cytology as primary test, medium‐high mortality and incidence rates, and medium vaccination coverage (Figure 2). It is important to highlight that most EU‐28 Countries are mainly located between the first and fourth quadrants with high income. On the contrary, the Countries outside of the EU‐28 are located between the second and third quadrant with upper‐middle income and lower‐middle income (Figure 3).

## 4 DISCUSSION

In 2015, 526.000 women developed CC worldwide and caused 239.000 deaths.^39^ The pap‐test screening programs, allowing an early diagnosis of precancerous lesions and a timely treatment of the same, have allowed to reduce the incidence of cervical cancer. Vaccination prevents precancerous lesions, reduces cancer and related treatments to eliminate precancerous lesions. Vaccination, acting much earlier in the history of disease development, prevents chronic infection resulting in pre‐cancerous lesions. Vaccination and screening programs are fundamental because they are potentially cost‐effective and allow decreasing incidence and mortality rates of CC.^40^ Screening, however, will remain fundamental for prevention of CC despite HPV vaccines.^41^ In fact, a factor that determines the differences in the incidence of CC among Countries is the screening coverage of the population.^7^

Monitoring HPV‐vaccination coverage is important to evaluate the performance of vaccination programs and the potential impact of HPV vaccine on cervical cancer. In fact, cervical cancer screening programs will need to be adjusted to the number of vaccinated people eligible for screening. However, despite the documented effectiveness of HPV vaccine, there is still an incomplete availability to this prevention action in the world population. Bruni et al^42^ showed high differences in number of women vaccinated according to gross income level countries; in fact, high‐quality primary and secondary cancer prevention is nearly always available in wealthy countries with gross national income (GNI) level.^42^ Moreover, higher income allows access to better resources and living standards and can increase the ability to maintain healthy behaviors.^43^ Syse and Lyngstand showed that high income is also related to higher survival rate.^44^

Our study shows that European Countries with higher income have higher screening and immunization coverage probably due to organized screenings starting before 1960 that determined low incidence and mortality rates, respect to those with lower‐middle income. High‐income countries have HPV screening test as the primary test and total or free partial charge HPV vaccination.

Eastern European and Asian Countries have lower‐middle income and show high incidence and mortality rates. These countries have an opportunistic screening with lower‐screening coverage and lower‐immunization coverage probably because HPV vaccine was introduced later. Globally, the coverage of vaccination is higher in countries with high income; by 2016, 71% of HI countries, 35% UMI countries, 8% of LMI countries, and 6% of LI countries had introduced the HPV vaccine.^45^

Only eight of the 70 countries who reported HPV vaccine introduction by the end of 2016, made the vaccine available to boys in addiction to girls (Australia, Austria, Barbados, Brazil, Canada, Italy, Switzerland, and the United States).^46^ According to Brisson et al,^47^ greater benefits can be acquired for both female and male by increasing HPV‐vaccination coverage among girls. In addition, vaccination of both sexes would be more equitable.^48^

In light of this, we would like to point out that: first, the strategy of including males in vaccination campaigns has, without a doubt, the function of reducing the circulation of the virus (herd‐effect) and the transmission of infection between the two sexes. It has also the advantage of countering the occurrence of HPV‐related diseases affecting male anatomic sites, such as the penis. Second, it is important to stress that both sexes have the same right to benefit from the advantages of anti‐HPV vaccination. In fact, according to European regulations, it is a right of every citizen to take advantage of disease prevention programs, where there is an effective means of prevention like the anti‐HPV vaccine. Third, a universal anti‐HPV vaccination program reduces the prejudices created around a female‐only vaccination, helping to reduce sociocultural barriers and thereby increasing acceptability and vaccination coverage.

Public health authorities should monitor the HPV‐vaccinated population in order to determine more precisely the effects on short‐ and long‐term incidence and mortality rates.

A useful scenario for crucial support to public health decision‐makers is the strength of our paper. On the other hand, a limitation could be that the data that came from low‐income countries must be considered with caution, both because they come from local registries (rather than the population‐based cancer registries used for the other countries) and because the International Classification Disease, 9th revision, codes are not always accurate.

## CONFLICT OF INTEREST

The authors declare no conflict of interest.
